# Perceived barriers and pathways to religious inclusion: UK Muslim clients' perspectives on faith in therapy

**DOI:** 10.1111/papt.70071

**Published:** 2026-05-01

**Authors:** Rumena Islam

**Affiliations:** ^1^ Doctoral College/Department of Psychology University of Bath Bath UK

**Keywords:** cultural competence, inclusion, Muslim clients, psychotherapy, qualitative research, religion, therapeutic relationship

## Abstract

**Objectives:**

This study explored how Muslim clients experience the minimisation or exclusion of religion in therapy and identified their recommendations for making therapeutic practice more inclusive of faith.

**Design:**

A qualitative design using reflexive thematic analysis was employed to examine participants' lived experiences and meaning‐making processes.

**Methods:**

Semi‐structured interviews were conducted with 25 Muslim adults in the UK who had received therapy in both NHS and private settings. Data were analysed inductively to capture individual and systemic influences shaping the minimisation or exclusion of religion in therapy.

**Results:**

Two overarching themes were developed: perceived barriers to discussing religion in therapy reflected therapists' limited confidence and knowledge, secular service frameworks and participants' resulting self‐censorship. Recommendations for facilitating religious inclusion captured participants' calls for faith‐sensitive competence, proactive engagement with religion in therapy and organisational change to legitimise faith within professional practice.

**Conclusions:**

Excluding religion risks marginalising clients' core identities and undermining therapeutic safety and ultimately compromising the effectiveness of treatment. Embedding religious competence within training, supervision and service policy is vital to ensure therapy is culturally responsive, ethically grounded and genuinely inclusive for clients of faith.

## INTRODUCTION

Religion is a central aspect of identity for many individuals worldwide, influencing values, coping strategies and meaning‐making processes (Koenig, 2012). In clinical settings, clients' religious beliefs can play a pivotal role in shaping their experiences of psychological distress and engagement with therapy (Dubey et al., 2025). However, despite growing recognition of the importance of culturally and spiritually competent care, research indicates that religion is often minimised, overlooked or excluded in therapeutic contexts (Istratii & Ali, 2023; Trusty et al., 2022). For clients for whom faith is integral, this omission may result in feelings of invisibility, disempowerment and dissatisfaction with psychological services, ultimately affecting therapeutic engagement and outcomes (Shafan‐Azhar et al., 2025).

Within mental health research, much attention has been given to therapists' perspectives on integrating religion into therapy (Pargament & Mahoney, 2005). Studies have highlighted barriers such as lack of training, fear of imposing personal beliefs or uncertainty about ethical boundaries (Islam & Chadwick 2025; Currier et al., 2023). However, comparatively little research has explored the clients' perspectives on how religion could be better integrated into therapy or why they perceive it is frequently excluded. This gap is particularly evident for minority religious populations, such as Muslims, whose experiences are shaped not only by general challenges in therapy but also by the broader socio‐cultural context, including experiences of discrimination, stereotyping and religious misunderstanding (Mahmud, 2024).

Emerging evidence suggests that clients are not passive recipients of therapy; they actively evaluate and interpret therapeutic processes and often have clear ideas about what might facilitate or hinder the inclusion of their religious identity (Knox et al., 2005). Understanding these perspectives is critical, both ethically and clinically, to support culturally responsive practice. Moreover, including clients' voices directly in research addresses a long‐standing critique of psychotherapy literature, which has historically privileged the therapist's perspective (Levitt et al., 2016). By focusing on clients' lived experiences, research can illuminate practical strategies for improving therapy, foster therapeutic alliance and reduce the risk of inadvertent marginalisation.

Theoretical frameworks such as cultural humility and religious competence provide guidance for integrating religion into therapy in a respectful and collaborative manner (Mosher et al., 2017). Cultural humility emphasises openness, self‐reflection and a commitment to understanding clients' cultural and religious contexts, rather than assuming expertise or imposing standardised approaches (Zhu et al., 2023). Religious competence similarly underscores the importance of therapists recognising, respecting, and, when appropriate, incorporating clients' faith in therapeutic work (Currier et al., 2023). While these frameworks provide principles, there remains limited empirical research examining the specific barriers clients perceive and the strategies they suggest for achieving meaningful integration of religion into therapy.

This paper builds on previous work exploring Muslim clients' experiences of religion being minimised or excluded in therapy (Islam & Chadwick, 2026). That study highlighted the emotional, relational and systemic impacts of religious exclusion, including feelings of invisibility, self‐censorship and challenges to religious identity. However, it did not examine potential strategies to support religious inclusion in therapy. The current study addresses this gap by focusing on participants' views regarding: (1) the reasons they perceive religion is not routinely integrated into therapy and (2) their recommendations for how therapeutic practice could better accommodate religious identity when it is important to clients. This approach shifts the analytical lens from exploring experiences of minimisation or exclusion, as in prior work, to exploring clients' solution‐focused perspectives, thereby providing actionable insights for clinicians and services seeking to deliver inclusive, culturally and religious responsive care. Religion’ and ‘spirituality’ are treated interchangeably in this paper to reflect participants' perspectives.

By centring clients' voices, this study contributes to the growing field of research on religion and psychotherapy by highlighting practical barriers, facilitators and client‐informed strategies for integrating faith into therapy. The findings have the potential to inform training, supervision and service development, ensuring that psychological care is both respectful of religious identity and aligned with best practice principles in culturally competent care. In doing so, this research not only addresses an empirical gap but also highlights an ethical gap: fulfilling the imperative for therapy to be inclusive, empowering and relevant to the diverse populations it serves.

## METHOD

This study was conducted and reported in accordance with the Consolidated Criteria for Reporting Qualitative Research (COREQ; Tong et al., 2007). Ethical approval for this study was granted by the relevant institutional ethics committee prior to data collection.

This study was conducted from a critical realist ontological stance which assumes that participants' experiences of therapy are grounded in reality while simultaneously shaped by broader social, cultural and religious contexts. This perspective allowed the research to explore both the objective elements of therapy and the subjective interpretations of how participants perceived the integration or exclusion of their religious identity (Braun & Clarke, 2013).

### Design

This study adopted a qualitative design using semi‐structured interviews to explore Muslim clients' perceptions of how religion is integrated within psychological therapy. This approach was selected to allow an in‐depth examination of participants' lived experiences and reflections on the ways in which their religious identity was acknowledged, minimised or excluded in therapy, as well as their perspectives on barriers and facilitators to its inclusion.

Semi‐structured interviews were chosen for their flexibility in enabling participants to share nuanced and personal accounts while ensuring coherence across key areas of inquiry. The qualitative design was underpinned by an experiential orientation, prioritising participants' subjective meanings and interpretations of their therapeutic experiences. To ensure methodological transparency and rigour, the study adhered to the Consolidated Criteria for Reporting Qualitative Research (COREQ; Tong et al., 2007).

### Participant eligibility

Given the study's focus on lived experiences, eligibility to participate was based on self‐reported experiences. To ensure participants met the criteria, a brief 10‐min screening interview was conducted, which included open‐ended questions to elicit initial qualitative accounts of their experiences without the constraint of predefined responses.

The inclusion criteria were as follows:

- participants were currently in therapy or had previously received psychological therapy from a mental health professional and perceived that their therapist had, at times, minimised or excluded their religion from therapy;
- participants self‐identified as Muslim and were residing in the UK;
- participants were aged 18 years or older; and
- participants were fluent English speakers.

Non‐English speakers were excluded due to the interpretative challenges associated with translation and the potential impact this could have on the authenticity and depth of qualitative analysis (Braun & Clarke, 2021a).

### Interview schedule

The semi‐structured interview schedule was designed to explore Muslim clients' experiences of therapy, including how their religion was acknowledged or excluded. The schedule was developed collaboratively with input from a Patient and Public Involvement (PPI) group consisting of 12 members of the UK Muslim community, including mental health professionals and individuals with lived experience of a range of mental health difficulties, representing diverse ages, genders and regions within the UK. Their feedback informed the cultural relevance, language and sensitivity of the questions to ensure accessibility and resonance with participants' experiences. For example, the term *“faith practices”* was preferred over *“religious rituals”*, as it was viewed as less clinical and more representative of participants' lived expressions of belief.

While the broader interview schedule examined experiences of religious minimisation or exclusion in therapy, the present study focuses on three specific questions that explored participants' perceptions of how religion could be integrated more effectively within therapeutic settings:

1. *How should mental health professionals approach the topic of religion in therapy with Muslim individuals?*
2. *Why do you think your therapist, consciously or unconsciously, excluded or minimised your religion?*
3. *Are there any parts of the therapy you found helpful where your faith was included?*

These questions, though collected alongside the broader dataset, were not included in the original analysis, as they represented a distinct area of inquiry. The current study therefore presents a focused thematic exploration of participants' views on the barriers to and facilitators of religious integration in therapy.

The interview schedule was piloted with a UK Muslim with prior therapy experience who met the study criteria. Feedback from this pilot was used to refine the flow, tone and accessibility of the questions. For instance, the question *“How should mental health professionals approach the topic of religion in therapy with Muslim individuals?”* was originally phrased as *“In what ways do you think clinicians should initiate discussions about Islam within therapeutic practice?”* Feedback from the pilot suggested that this earlier version sounded overly academic and positioned the participant as an evaluator of clinical competence. It was reworded to create a more collaborative tone, encouraging participants to share their views as lived experts rather than as critics of professional practice.

### Recruitment

Participants were recruited via Muslim organisations, local mosques and social media platforms across the UK. Individuals expressing interest were asked to contact the researcher directly, after which eligibility was assessed through a brief screening interview covering age, religion, English proficiency and experiences of religion being minimised or excluded in therapy. Eligible participants were then provided with detailed study information and consent forms prior to participation.

To acknowledge participants' time and contribution, a £10 stipend was provided at the conclusion of the interview. In line with the flexible and interpretive nature of reflexive thematic analysis (RTA; Braun & Clarke, 2021b), no predetermined sample size was imposed. Recruitment of 25 participants was deemed sufficient to generate rich, meaningful data aligned with the study aims.

### Screening for participant eligibility

To safeguard the integrity of the dataset and ensure participants met inclusion criteria, a systematic screening protocol was implemented, informed by best practice recommendations for online research recruitment (Ridge et al., 2023; Santinele Martino et al., 2024). Potential participants expressing interest via email were assessed for eligibility before follow‐up. Applications were excluded if they suggested a primary interest in monetary compensation, displayed copied text from recruitment materials or exhibited inconsistent or minimal information regarding their therapy experiences.

Eligible participants were then invited to complete a brief 10‐min screening interview, during which self‐reported experiences of therapy, engagement with religion in therapy, age and English language proficiency were confirmed. Clarifying questions were used to assess narrative coherence and ensure participants' experiences were authentic. This process ensured that the study captured rich, credible qualitative data from individuals genuinely meeting the inclusion criteria, consistent with principles of methodological rigour in qualitative research (Johnson et al., 2020).

### Procedure

Participants were provided with an electronic information sheet and a written consent form, with the opportunity to ask questions about the study before deciding to participate. Eligibility was confirmed prior to scheduling the interview.

Interviews were conducted remotely via Microsoft Teams between February and June 2024, each lasting around 45–60 min. The interviews were divided into two segments: the first focused on collecting demographic information and the second explored participants' experiences of therapy, emphasising barriers to the integration of religion and recommendations for improvement. Participants were asked to provide demographic information including age, gender and racial/ethnic background. Age was included to provide basic context for the sample, while gender and racial/ethnic background were collected to allow consideration of diversity and potential differences in experiences and to situate participants' accounts within broader social and cultural contexts.

Microsoft Teams' recording and transcription functions were used to create initial transcripts, which were subsequently reviewed for accuracy by the lead researcher (RI) and a research assistant (EV). After each interview, participants were debriefed and thanked for their time. Transcripts were anonymised prior to analysis.

RTA (Braun & Clarke, 2021a) was applied, with the lead researcher maintaining a reflective journal throughout the process to capture initial impressions, emerging insights and potential biases, thereby supporting reflexivity and analytical transparency.

### Data analysis

Data were examined using RTA (Braun & Clarke, 2019; Braun & Clarke, 2021a, 2021b; Braun et al., 2023), selected for its flexibility in capturing complex, nuanced qualitative data. RTA enables the identification, interpretation and reporting of patterns (themes) within the data, providing insight into participants' experiences while recognising the researcher's active role in meaning‐making (Braun & Clarke, 2019). The six‐phase framework outlined by Braun and Clarke (2019) guided the analytic process (see Table S1 for detailed implementation).

Coding was conducted at both semantic and latent levels, allowing for the exploration of both explicit content and underlying meanings within participants' accounts. An inductive approach was applied to privilege participants' perspectives and ensure that themes emerged naturally from the data rather than being constrained by pre‐existing theories (Braun & Clarke, 2021a). Some deductive elements were incorporated, guided by the research question and prior literature on negative therapy experiences among Muslim clients, highlighting the importance of balancing inductive insight with theoretical awareness (Joffe et al., 2012).

Trustworthiness was carefully maintained throughout the analytic process. A detailed audit trail documented all methodological decisions, supporting transparency and reproducibility. Reflexive practices, including journaling and peer discussions, were employed to monitor the influence of researcher positionality, particularly the primary investigator's (RI) identity as a Muslim, on data interpretation. Credibility was enhanced through in‐depth engagement with the transcripts and collaborative dialogue with the research team, ensuring multiple perspectives informed theme development. Reflexive journaling and peer debriefing also facilitated critical examination of assumptions, biases and emotional responses, contributing to the overall confirmability and robustness of the study's conclusions (Ahmed, 2024; Megheirkouni & Moir, 2023).

### Researcher context

The primary researcher (RI) led recruitment and data collection, supported by the research supervisor (PC) and a research apprentice (EV). RI is a British Asian Muslim female and a recently qualified clinical psychologist. Their personal and professional experiences informed the choice of research topic and sensitivity to participants' accounts. PC is a White British male, with over 30 years of experience as both a clinical psychologist and researcher; his religious beliefs are not known to the primary researcher. EV is a White British Christian female undergraduate participating through a university research apprenticeship scheme. Additional insight was provided by a Turkish British female Expert by Experience, with 20 years' experience and 5 years as a research assistant for a Muslim mental health charity, who reviewed the themes and findings. This collaboration helped to mitigate potential researcher bias, offering alternative perspectives on the interpretation and analysis of participants' accounts.

## RESULTS

### Participants

Twenty‐five Muslim clients participated in this study, including seventeen females and eight males. Participants ranged in age from 18 to 56 years, with an average age of 30. The sample comprised individuals from diverse ethnic backgrounds, with seventeen identifying as British Asian, four as Black British and four as Arab. Participants were geographically dispersed across the United Kingdom, with seven living in London, six in the Northwest, five in the Northeast, two in the Southeast, one in the West Midlands, one in the East Midlands and one in Wales.

All participants had accessed psychological therapy within the past 5 years through NHS services (*n* = 15), private practice (*n* = 8) or university counselling (*n* = 2). Presenting difficulties included anxiety, depression, trauma and psychosis. Some participants described long‐standing mental health difficulties, while others sought therapy in response to specific life events or situational stressors.

Although the study did not aim to compare service types or regions, some variation emerged. NHS and university clients described therapy as rigid and ‘tick‐box’, while private clients noted greater flexibility. Nonetheless, participants across all services reported religion being minimised or excluded, indicating that service context may shape delivery but does not prevent marginalisation of religious identity.

**FIGURE 1:**
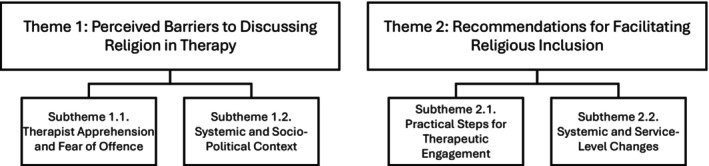
A thematic map depicting final themes, sub‐themes and their relationship.

RTA of the interviews generated two main themes capturing participants' experiences and perspectives (see Figure 1). The first theme, perceived barriers to discussing religion in therapy, reflects participants' views on why religion was minimised or excluded. The second theme, recommendations for facilitating religious inclusion, encompasses participants' suggestions for how therapists and services could better integrate religion into therapeutic practice.

### Theme 1: Perceived barriers to discussing religion in therapy – “*It Felt Like Faith Was Off‐Limits*” (P9)

This theme reflects participants' accounts of the main challenges that contributed to religion being minimised, excluded or overlooked in therapy. Barriers emerged from perceived limitations in therapists' knowledge and confidence, systemic constraints within mental health services and participants' own tendencies towards self‐censorship.

A prominent barrier reported by participants was the perception that non‐Muslim therapists lacked sufficient understanding of Islam. This often manifested as dismissiveness, misinterpretation or minimisation of religious concerns, leaving participants feeling that their faith was undervalued within therapeutic interactions. One participant reflected that their therapist *“did not have any knowledge”* about Islam, suggesting that this explained their reluctance to engage in discussions about faith (P1). Another participant noted that attempts to share the significance of reading the Quran were *“really quickly dismissed, but it was like a hobby or something and not a lot of attention was paid towards that”* (P7). Similarly, another participant described feeling that their therapist *“didn't care about my religion even though it was important to me”* and would *“brush over it”* when they tried to explain aspects of their faith (P19).

Six participants linked therapists' lack of engagement with religion to the structured nature of certain therapeutic modalities, particularly cognitive behavioural therapy (CBT). They described these approaches as highly procedural, focusing on symptom management and step‐by‐step techniques, which left little space for clients to bring in personal or faith‐related perspectives. This rigid framework made it difficult for participants to explore how their religious beliefs informed their experiences of distress.

*It felt very structured, like ticking boxes ‘is that one managed, OK next thing.’ There was no space for me to talk about how my faith shapes my anxiety or coping*. (P8).

Participants also described the emotional labour involved in feeling responsible for educating therapists about Islam. They reported that explaining core aspects of their faith or clarifying its relevance to their mental health became an additional burden, on top of managing their distress within therapy.

*I didn't want to have to educate and explain. It was exhausting trying to teach them about my religion while also talking about my problems*. (P15).

### Subtheme 1.1. Therapist apprehension and fear of offence

Participants frequently interpreted therapists' reluctance to engage with religion as stemming from apprehension or fear of causing offence. Many described this as a lack of confidence or skill in addressing religious topics, particularly Islamic beliefs and practices, within therapy sessions. This uncertainty often meant that therapists would avoid exploring faith altogether, leaving participants feeling their core identity was overlooked or treated as secondary to their mental health concerns.

*There was a lack of understanding from the therapists in terms of how to integrate religion into therapy. I could tell they weren't sure what to do with it, so it just felt like my faith wasn't welcome*. (P2).

*I could sense that my therapist didn't know how to handle anything about religion. It made me hesitant to say anything about my faith at all*. (P23).

Some participants suggested that therapists' hesitancy stemmed from not knowing how to respond appropriately when religion was mentioned, resulting in silence rather than meaningful engagement. This silence was interpreted as a subtle dismissal or discomfort with faith‐related discussions:

*They didn't know what to respond, so they didn't respond anything. It made me feel like I was talking about something that wasn't supposed to be discussed*. (P4).

Others described this reluctance as fear‐based, where therapists seemed anxious about saying the wrong thing or damaging the therapeutic alliance. Participants highlighted that even well‐intentioned therapists could inadvertently communicate disapproval or distance through non‐verbal cues or lack of follow‐up:

*The fear of not knowing the right thing to say. Fear of not understanding what needs to be said. Fear of, you know, causing a rupture in the relationship. It was like walking on eggshells every time I tried to bring it up*. (P6).

This apprehension often resulted in minimal engagement or superficial treatment of religious content. Even when participants attempted to connect their experiences to their faith, therapists sometimes appeared disengaged, leaving participants feeling isolated or misunderstood:

*When I tried to connect my experiences to my faith, my therapist looked confused and didn't ask any follow‐up questions. It felt like I was speaking a different language, and my faith wasn't being heard at all*. (P11).

In other cases, therapists actively avoided discussing religion to prevent eliciting negative responses or potential conflict, which reinforced the perception that faith was unwelcome in therapy.

*They didn't engage with my religion because it might make me feel negative. I started to feel like my faith was almost not allowed to be mentioned*. (P7).

### Subtheme 1.2. Systemic and socio‐political context

Participants situated the barriers to discussing religion within broader systemic and socio‐political contexts, highlighting that these challenges extended beyond individual therapists to the frameworks and structures of mental health services. Therapy was often described as embedded within secular, Western‐centric models that prioritised symptom management and procedural approaches, leaving little room for faith‐based considerations.

*Therapy feels built for a Western audience like it's not made for people who see life through faith*. (P12).

Several participants emphasised that therapeutic models often relied on a “one size fits all approach,” which failed to accommodate the nuanced ways in which religion shapes clients' experiences of distress and coping. This framework left participants feeling that their religious identity was peripheral rather than central to their mental health journey.

*It felt like therapy had a checklist for everyone a standard set of steps but there was no space to talk about my faith or how it relates to my struggles*. (P17).

Professional training was also highlighted as a contributing factor for those from a mental health background (*n* = 5). Participants reflected that religion was largely overlooked in education and clinical preparation, despite coverage of other cultural and diversity topics. This omission contributed to therapists' lack of confidence and reinforced structural exclusion.

*In my mental health training, we covered culture and diversity, but religion was really missed out it's like it doesn't count*. (P22).

Wider societal factors, including negative media portrayals and prejudice against Muslims, were also reported as contributing to participants' apprehension. One participant described entering therapy *“bearing the weight of societal prejudices about Islam”* and feeling pressured to be a *“good representative”* of their faith (P7). Another participant expressed that they felt the need to convince the therapist that they *“did not fit the stereotypes you see in the media”* (P15).

Taken together, these reflections indicate that barriers to discussing religion were not simply due to individual therapist limitations. Instead, they were embedded in structural, cultural and societal contexts that collectively signalled that faith was a sensitive or unwelcome topic. This systemic and socio‐political climate contributed to participants' self‐censorship and limited opportunities for religion to be meaningfully integrated into therapy.

*After a while, I just stopped bringing it up. I didn't want to make things uncomfortable, and I could sense the wider pressures around how Islam is viewed in society*. (P9).

### Theme 2: Recommendations for facilitating religious inclusion “*Making Space for Faith in Therapy*” (P22)

Participants reflected on the importance of recognising religion as a legitimate and integral aspect of clients' identities within therapy. They described how excluding or minimising faith could leave clients feeling unseen, invalidated or hesitant to share experiences that were deeply meaningful. Creating a safe space for religious expression was seen as requiring active recognition of the personal, cultural and spiritual significance of faith and understanding how it shapes individuals' lives. Integrating religion into therapeutic practice was viewed not simply as an optional consideration, but as central to delivering culturally responsive and psychologically attuned care.

*When I couldn't bring my faith into sessions, I felt like a part of me was missing. Therapy didn't feel like it was really for me*. (P21).

*It felt like my faith didn't belong in therapy, so I held back from sharing things that were really important to me*. (P22).

Participants highlighted that clients' comfort in discussing faith depended on therapists signalling that religion is legitimate and welcome, and on services fostering an environment where conversations about faith are considered normal and professionally supported. They emphasised that when therapists approach faith with curiosity, openness and respect, rather than avoidance or assumption, it validates clients' experiences and acknowledges the ways faith can support coping and resilience.

*My faith isn't a problem to solve it's part of how I cope. Therapists need to see that and work with it, not around it*. (P20).

### Subtheme 2.1. Practical steps for therapeutic engagement

Participants identified several practical strategies therapists could adopt to integrate religion effectively and sensitively into therapy. Acquiring foundational knowledge about Islam was viewed as essential for confident engagement. This could be gained through accessible methods such as brief independent research.

*Just like an hour of Googling could give a basic understanding*. (P1).

Experiential learning was also recommended, including opportunities to hear directly from clients or carers with lived experience of faith‐informed mental health challenges. Participants emphasised that this exposure deepens understanding of how religion shapes coping, resilience and meaning‐making, helping therapists integrate it appropriately into sessions.

*I think it would really help if therapists could hear from people like me or carers who've actually lived through this*. (P12).

Participants described a holistic and contextualised approach as central to effective engagement. This involved recognising faith as a source of resilience rather than a complicating factor and exploring ways to link therapeutic strategies with religious practices, such as prayer [dua], to support coping and recovery.

*I wanted a therapist who could link the two, understanding my coping strategies alongside my prayers and religious practices*. (P14).

Therapists were encouraged to avoid assumptions and to treat each client as an individual, recognising the diversity of experiences, religious expression and personal levels of religiosity. Participants highlighted that Islam encompasses a wide range of practices and interpretations, and what is meaningful to one client may not apply to another. This approach involves adopting a curious and open stance, asking questions rather than making presumptions, and validating each client's perspective.

*Even within Islam, people do things differently. You can't assume everyone follows the same rules or practices*. (P9)

*You can belong to a certain faith or culture, but each person experiences it differently. Therapists need to see that*. (P23).

Practical strategies also included proactively asking about faith, demonstrating openness to religious topics and integrating discussions of spirituality within coping plans and therapeutic goals. Participants emphasised that these actions signal respect, legitimise faith‐related content and reduce clients' self‐censorship, fostering a stronger therapeutic alliance.

*Even just being asked about my faith made me feel like it mattered and that my therapist really wanted to understand me*. (P18).

### Subtheme 2.2. Systemic and service‐level changes

Quality of culturally sensitive care was seen as inseparable from workforce diversity. Many participants advocated for the employment and training of more Muslim therapists to create safer, more relatable spaces where clients would not feel the need to repeatedly explain or justify their faith. Representation was viewed not only as a matter of inclusion but also as a source of validation and trust. Several participants extended this recommendation to the organisational hierarchy, emphasising the importance of having Muslims in senior or decision‐making positions. They felt that when leadership reflects the communities being served, it signals genuine commitment to inclusion and ensures that policies and service priorities are informed by lived experience. One participant explained that seeing Muslims in leadership “would show that our faith and our perspective actually matter within the system” (P15).

*It's not just about having Muslim therapists on the ground we need Muslims in higher places making decisions, setting the tone for what inclusion actually looks like*. (P19).

*Having more Muslim therapists or staff who understand faith would make a huge difference. But it also needs to be part of the service culture, not just individual therapists*. (P10).

Participants also suggested that senior leaders and policymakers embed religious inclusion into organisational culture and practice standards. They emphasised that genuine change requires commitment beyond individual therapists, calling for systemic accountability and leadership‐driven initiatives. This included incorporating religion into equality, diversity and inclusion (EDI) frameworks, service policies and embedding modules on religious competence into core clinical training. Participants felt that when faith inclusion is endorsed and modelled at an organisational level, therapists are more likely to feel confident and legitimised in bringing these discussions into their clinical work. Without such top‐down reinforcement, religion risks remaining marginalised or treated as a personal therapist preference rather than an ethical and professional responsibility.

*Every therapist should learn the basics of religion in therapy as part of their core training*. (P5).

*It shouldn't depend on whether you get a good therapist or not services themselves need to make it normal to talk about faith*. (P13).

Client choice was another key priority, with participants highlighting the value of being able to select therapists who demonstrated understanding and sensitivity towards Muslim clients' cultural and religious contexts. While some expressed a preference for Muslim therapists, many clarified that what mattered most was working with practitioners who were knowledgeable, respectful and confident in engaging with faith‐related discussions. This sense of choice was seen as fundamental to building trust and creating a therapeutic space where clients could bring their full identities without fear of judgement or misunderstanding. However, participants described how such options were often limited, inconsistently available or poorly communicated. Some reported being unaware that they could express preferences for therapists with relevant cultural competence, while others felt that doing so might be perceived as unreasonable or demanding.

*It's not that my therapist had to be Muslim they just needed to understand how to work with someone like me, where faith is such a big part of who I am*. (P16).

*It would really help if I could ask for a Muslim or culturally aware therapist. Right now, that option isn't clear or even available*. (P14).

Holistic service models were strongly encouraged, integrating spiritual, religious and psychological dimensions of care to reflect the interconnected nature of clients' wellbeing. Participants described wanting services that recognised faith as an integral part of mental health rather than a separate or peripheral concern. This approach, they argued, would help bridge the gap between clinical and community support, fostering a more inclusive model of care.

*For me, my faith isn't separate from how I cope or understand things. Therapy should work with that, not around it*. (P20).

Participants proposed a range of ways to operationalise this integration, including closer collaboration between mental health professionals and faith leaders, the development of faith‐informed therapy options and co‐facilitated sessions where religious leaders work alongside therapists to address clients' concerns from both psychological and religious perspectives. Such collaboration was seen to enhance clients' comfort, promote cultural safety and normalise the inclusion of faith‐based discussions within therapeutic spaces.

*If there was someone there who understood both the therapy side and the faith side I think I'd trust the process more*. (P11).

Greater sensitivity to accessibility was also emphasised, with participants highlighting the need for therapy services to be more flexible, responsive and attuned to the practical realities of clients' lives. Accessibility was described not only in terms of availability and waiting times but also in the way therapy was structured, paced and delivered. For some, standard session formats and rigid attendance expectations felt incompatible with the fluctuating emotional, spiritual or family demands that shaped their wellbeing. As one participant explained,

*Sometimes it feels like therapy moves too fast like there's a checklist to get through. I needed more space to process things at my own pace*. (P13).

Participants proposed that services adopt more flexible approaches, such as offering varied session lengths, hybrid delivery options (including online sessions for those balancing family or work commitments) and consideration of religious observances, such as prayer times or Ramadan, when scheduling appointments.

*Even small things like not scheduling during prayer time show respect and make therapy more doable*. (P10).

Participants emphasised that meaningful inclusion of religion in therapy requires not only individual therapist awareness but also institutional accountability. One of the key recommendations was the development of culturally and spiritually sensitive outcome measures that reflect clients' faith‐related goals and values. Standardised tools were often perceived as limited, with one participant noting that “the forms don't really capture what healing means for someone whose faith is part of that process” (P2). Another explained that progress for many Muslim clients might be reflected in “feeling closer to God or finding peace through prayer,” rather than symptom reduction alone (P16).

*It would help if therapy measured progress in ways that include my faith, not just symptoms. My spiritual goals matter too*. (P24).

To sustain these changes, participants also called for the creation of supervision and peer consultation spaces where therapists could explore the complexities of integrating religion in practice without fear of judgement. Participants expressed that therapists might avoid discussing religion due to “not wanting to say the wrong thing,” suggesting that structured, faith‐informed reflective practice could normalise these conversations (P4).

*Supervision should be a place where you can talk about faith just like you talk about race or gender it shouldn't be excluded*. (P7).

*Supervision should let therapists talk about religious issues without feeling judged, so they can learn how to handle it better*. (P18).

Participants also highlighted the need for clear organisational guidance to support therapists in navigating discussions about faith confidently and ethically. Many felt that the absence of explicit policies left therapists uncertain about whether raising religion was appropriate, leading to avoidance by default. Developing service‐wide guidelines was therefore seen as essential, not only to legitimise conversations about faith, but to signal to clinicians that these topics fall within the scope of good therapeutic practice rather than outside of it.

*If there were clear guidelines on how to talk about religion, I think therapists would feel more confident, and clients like me wouldn't feel like it was taboo*. (P23).

*Having some kind of official guidance would make it easier for therapists to include faith without worrying about doing something wrong*. (P19).

Such guidelines, participants suggested, could offer practical frameworks, examples of sensitive language and prompts for exploration, ensuring that clinicians feel supported rather than anxious when approaching religious themes. This clarity was viewed as particularly important in multicultural contexts, where therapists may fear causing offence or overstepping boundaries. With appropriate guidance, participants believed therapists would be more likely to engage meaningfully with clients' faith, rather than relying on silence or avoidance due to uncertainty.

## DISCUSSION

### Summary of results

This study explored Muslim clients' experiences when religion was minimised or excluded in therapy, highlighting how such exclusion shaped their sense of visibility, safety and engagement in the therapeutic process. The findings contribute to a growing body of literature demonstrating that religion and spirituality are often underexplored within therapy, despite their salience in clients' meaning‐making and coping (Islam & Chadwick, 2025; Knox et al., 2005). Across participants' accounts, religion emerged not as an optional add‐on to psychological work, but as a deeply embedded aspect of identity that required active recognition and inclusion. The themes, perceived barriers to discussing religion in therapy and recommendations for facilitating religious inclusion, capture both the difficulties Muslim clients encounter and their vision for what more inclusive, faith‐sensitive therapy could look like.

Distinct from prior research that has primarily focused on the exclusion of faith in therapy (Islam & Chadwick, 2026), this study extends the discourse by centring clients' own recommendations and solution‐focused perspectives on how religious inclusion can be meaningfully achieved. By shifting focus from describing barriers to exploring client‐informed strategies, the study provides a novel contribution to understanding how faith can be actively integrated into therapeutic practice.

#### Theme 1: Perceived barriers to discussing religion in therapy

Participants described therapy as a space where religion often felt off‐limits. These findings echo previous research indicating that therapists' apprehension, secular training contexts and societal narratives about Islam can inhibit open discussions about faith (Knox et al., 2005; Meer & Modood, 2019). Consistent with the concept of therapist avoidance (Byrne et al., 2021), participants perceived that therapists' reluctance stemmed from fear of causing offence or uncertainty about how to respond. This avoidance not only silenced religious content but also risked reproducing subtle microaggressions, whereby clients' core identities were minimised or pathologised through omission (Trusty et al., 2022). Parallels can be drawn with research on racially minoritised clients, who report that disclosure of racial trauma is frequently minimised or silenced in therapy (e.g., Samuel & Simonds, 2025), suggesting that suppression of core aspects of identity may be a broader issue in therapeutic practice.

The emotional labour participants described having to educate their therapists about Islam reveals a power asymmetry in the therapeutic encounter. Such dynamics align with research on cultural humility, which emphasises the need for therapists to approach difference with openness and self‐awareness rather than presumed expertise (Mosher et al., 2017). However, when humility is not paired with curiosity and engagement, it can slip into passivity, leaving clients responsible for bridging cultural gaps. For many Muslim participants, this burden compounded their sense of being “othered” within a secular mental health framework that privileges Western, individualistic understandings of distress. Related qualitative research has also identified a similar phenomenon in other contexts: for example, racially minoritised clients discussing experiences of racism in therapy often described *“holding the burden”*, feeling responsible for educating therapists and carrying additional emotional weight (Samuel & Simonds, 2025).

Participants' accounts also pointed to broader systemic and socio‐political constraints. The perception that therapy operates within a secular, Western model mirrors critiques from cross‐cultural and decolonial psychology (Fernando, 2021), which argue that dominant therapeutic paradigms often marginalise non‐Western worldviews. Several participants linked their discomfort to societal Islamophobia and negative media portrayals, describing a sense of vigilance in having to present themselves as “good Muslims.” These findings highlight how broader societal discourse seeps into the therapy room, shaping both clients' willingness to disclose and therapists' comfort in engaging with faith. Such insights emphasise that religion cannot be understood purely at the individual level; it is deeply intertwined with the socio‐cultural and political context in which therapy takes place.

#### Theme 2: Recommendation for facilitating religious inclusion

In contrast to these barriers, the theme recommendations for facilitating religious inclusion captured participants' hopes for therapy to become a space where faith is not only tolerated but meaningfully integrated. Participants viewed inclusion as both a relational and systemic endeavour. On the relational level, they described the importance of therapists signalling that faith is welcome, approaching it with curiosity, respect and genuine interest. This aligns with frameworks of spiritual competence, which advocate for therapists to develop awareness, knowledge and skills in addressing clients' religious and spiritual identities (Vieten et al., 2013). Participants' emphasis on proactive engagement, such as asking about faith early in therapy, suggests that even small gestures can powerfully legitimise clients' religious identities and reduce self‐censorship.

At the systemic level, participants called for structural and organisational reform. These included embedding faith within equality, diversity and inclusion (EDI) policies, developing clear service guidelines and integrating religious competence into clinical training and supervision. Such recommendations parallel findings from studies with other minority faith groups (Hodge, 2020), underscoring that inclusion cannot depend solely on individual therapist motivation. Participants also advocated for greater workforce diversity, noting that seeing Muslim clinicians and leaders in services signalled authenticity in inclusion efforts. This representation was viewed not only as culturally validating but also as critical to shifting organisational culture and challenging the implicit secular norms that shape service delivery.

Participants' desire for holistic service models where psychological dimensions are seen as interconnected reflects a move towards more integrative paradigms of care. The inclusion of faith leaders, faith‐informed therapy options and religiously sensitive measures of progress illustrates a vision for mental health care that honours clients' plural ways of knowing and healing. This approach resonates with community and liberation psychology perspectives, which advocate for interventions grounded in cultural and religious realities rather than imposed from dominant epistemologies (Watkins & Shulman, 2008).

#### Cross‐cutting insights: Self‐censorship and epistemic invalidation

This study extends current understandings of the intersection between religion, identity and therapy by illustrating how the exclusion of faith can constitute a form of epistemic invalidation the dismissal or devaluation of clients' ways of knowing and meaning‐making (Fricker, 2007). For Muslim clients, religion was not peripheral to identity; it was the lens through which life, suffering and recovery were understood. When therapy ignored this dimension, participants experienced a disconnection between self and treatment, echoing Klussman et al.'s (2021) argument that neglecting clients' spirituality may hinder therapeutic alliance and outcome.

The findings also illuminate how self‐censorship – the conscious or unconscious suppression of one's religious expression to avoid perceived invalidation or discomfort operates as a coping strategy in response to therapist avoidance or systemic marginalisation. These dynamics echo work on minority stress (Frost & Meyer, 2023) and extend it to the therapeutic setting, demonstrating how Muslim clients may regulate their expression of faith to protect themselves from invalidation. Conversely, the study highlights the empowering potential of spiritually inclusive practice, which recognises faith as a source of resilience and meaning. This aligns with existential and humanistic frameworks that position authenticity and identity coherence as central to psychological growth.

Collectively, these insights contribute to a theoretical understanding of how epistemic invalidation and self‐censorship function as interrelated processes that both reflect and reproduce systemic inequities within therapeutic encounters.

### Strengths and limitations

A notable strength of this study is its amplification of Muslim clients' voices, addressing a critical gap in psychological research where the intersection of faith and therapy is often overlooked. The use of in‐depth qualitative interviews facilitated rich, nuanced insights into both the barriers experienced and practical recommendations for faith‐inclusive practice. By including participants across NHS, private and university services, the study captures a diverse range of therapeutic contexts, enhancing the transferability of findings. The integration of PPI consultation and expert by experience input strengthened the study's methodological rigour, while the application of RTA ensured an in‐depth, transparent engagement with the data. Collectively, these elements position the study to provide meaningful guidance for both clinical practice and service‐level policy reform.

Several limitations should be considered. The sample comprised English‐speaking participants with access to digital platforms, which may have excluded linguistically or digitally marginalised individuals. Consequently, the findings may reflect a particular segment of the Muslim population those already engaged with mainstream therapy rather than the full diversity of Muslim clients in the UK.

The study did not differentiate between therapeutic modalities, theoretical orientations or therapist characteristics, limiting the ability to examine whether particular approaches or therapist factors influenced the silencing of religious or spiritual content. Approaches such as CBT, person‐centred therapy and psychodynamic psychotherapy may vary in openness towards religious exploration and future research could investigate how specific modalities influence the inclusion or avoidance of faith. In addition, the absence of demographic data on participants' racialised identities means that intersectional experiences of clients could not be explored. Together, these factors constrain the generalisability and depth of interpretation regarding how therapy practices may interact with religion, race or other intersecting aspects of identity.

Data relied on retrospective, self‐reported experiences, which are interpretive and may be influenced by memory or social desirability biases. Participant validation (e.g., member checking) was not employed; instead, trustworthiness was maintained through a transparent analytic process, detailed audit trail and regular supervision.

Finally, participants' accounts were embedded within broader socio‐political contexts, including Islamophobia, secular biases within health care and experiences of marginalisation which provided an important backdrop to their expectations of, and experiences, within therapy. While the study cannot fully disentangle systemic influences from individual therapist behaviour, the findings underscore the need for therapists to critically consider how broader societal forces shape therapeutic encounters. Islamophobia, secular norms within health care, and clinicians' own unexamined biases, privileges and assumptions may influence whether religious identities are recognised or marginalised in therapy. Acknowledging and actively reflecting on these dynamics is therefore a key professional responsibility in providing culturally responsive and inclusive psychological care. Future research should examine therapists' experiences, service policies and organisational conditions that facilitate faith inclusion.

### Clinical implications

The findings underscore the need for mental health practitioners to move beyond cultural competence towards religious humility, an ongoing process of learning, reflection and openness to clients' worldviews. Therapists should cultivate openness and a willingness to learn about clients' faith traditions and belief systems and be encouraged to explicitly invite discussion of faith or worldview early in therapy where it is relevant to the client. Training programmes should integrate modules on religious and spiritual competence, including case examples, ethical considerations and reflective exercises to build confidence in engaging with faith sensitively.

At the service level, faith inclusion should be formalised through clear guidelines embedded within EDI frameworks, with religious competence integrated into supervision, professional development and standardised outcome measures. This aligns directly with participants' recommendations in the current study, who emphasised that without organisational support, therapists often feel uncertain about engaging with faith, leaving clients' religious identities marginalised. For example, BPS, HCPC and BACP frameworks emphasise cultural competence, diversity and inclusivity, yet religion is rarely foregrounded as a core competency or detailed within accreditation standards for clinical psychology programmes. Policy‐level implementation would ensure consistency across diverse services, reduce reliance on individual therapist initiative and promote equity for clients for whom faith is central to wellbeing. This approach moves religious competence from an optional consideration to an integrated aspect of professional practice, consistent with broader commitments to cultural responsiveness and client‐centred care.

Future research could evaluate the effectiveness of organisational policies and training interventions on therapist confidence, client engagement and therapy outcomes. Comparative studies could examine differences across service types (NHS, private, university) or therapeutic modalities and explore whether similar barriers and facilitators exist for clients from other religious backgrounds, supporting the development of broadly applicable, evidence‐based, faith‐inclusive practice.

Supervisors also play a pivotal role in legitimising discussions about religion, supporting therapists in processing uncertainty and modelling inclusive practice (Aten & Hernandez, 2004). Supervisory training and continuing professional development should therefore include guidance on engaging with religion and belief in therapy, helping clinicians develop confidence, reflect on potential biases and integrate clients' faith identities into therapeutic work where relevant. Services should also consider evaluating outcomes that reflect clients' spiritual as well as psychological wellbeing, recognising that healing may encompass spiritual reconnection or growth in addition to symptom reduction.

Future research could build on these findings in several ways. Beyond exploring therapists' experiences of integrating religion, studies could examine the effectiveness of specific organisational policies or training interventions designed to improve religious inclusion. Comparative studies could investigate how different therapeutic modalities and service contexts (e.g., NHS, private practice, university services) impact faith inclusion. Additionally, research could explore the experiences of clients from other religious backgrounds to understand whether similar barriers and facilitators operate across faith groups, supporting the development of inclusive, culturally responsive mental health services.

Workforce diversity was highlighted by participants as both symbolically and practically important. Seeing Muslim clinicians and leaders in services can signal authenticity in inclusion efforts, challenge implicit secular norms and foster an organisational culture in which faith‐sensitive practice is valued and supported.

Collectively, these recommendations point towards a model of therapy in which religious and spiritual identity is integral rather than peripheral, supporting clients to engage authentically while reducing the burden of self‐censorship.

While this study focused on Muslim clients' experiences, the methodological approach could be adapted to explore how other religious groups experience inclusion or exclusion in therapy. Future research could replicate the design with participants from diverse faith backgrounds, using similar semi‐structured interviews and RTA to identify barriers and facilitators specific to different religions. Such work would support the development of evidence‐based, religion‐sensitive guidelines and interventions applicable across therapeutic contexts, ensuring that client‐centred care genuinely accommodates spiritual as well as psychological needs.

## CONCLUSION

Extending previous work, this study centres clients' own recommendations and solution‐focused perspectives on how religious inclusion can be meaningfully achieved. The findings emphasise the need to embed faith within Equality, Diversity and Inclusion frameworks and integrate religious competence into clinical training, supervision, and policy. Applying the lens of epistemic invalidation and self‐censorship, this study underscores that acknowledging clients' faith is essential for equitable, person‐centred psychological care in an increasingly diverse society.

## AUTHOR CONTRIBUTIONS

**Rumena Islam:** Conceptualization; methodology; investigation; writing – original draft; writing – review and editing; project administration; formal analysis; resources; data curation.

## CONFLICT OF INTEREST STATEMENT

The author declares no conflicts of interest.

## Supporting information


**Table S1.** Application of reflexive thematic analysis.

## Data Availability

The data that support the findings of this study are available on request from the corresponding author. The data are not publicly available due to privacy or ethical restrictions.
